# The effects of electricity network development besides routine malaria control measures in an underdeveloped region in the pre-elimination phase

**DOI:** 10.1186/s12936-016-1273-y

**Published:** 2016-04-18

**Authors:** Shahrokh Izadi

**Affiliations:** Health Promotion Research Centre, School of Public Health, Zahedan University of Medical Sciences, Zahedan, P.O. Box 98155-759, Iran

**Keywords:** Malaria, Epidemiology, Development, Spraying, Electricity

## Abstract

**Background:**

The main purpose of this study was to investigate the effects of electricity network development on malaria transmission. The study was performed in the rural areas of three districts in Sistan-va-Baluchestan Province, Iran.

**Methods:**

From the mentioned districts, 122 rural communities were selected. The data of the years 2005–2009 were collected retrospectively from data banks of the district health centres and the offices of the local electricity network. Fixed and random effects panel data regression models were fitted to determine the effects of electrification and other variables on malaria transmission during the elimination phase.

**Results:**

It seems that access to electricity of rural communities, if not harmful, has no obvious effect on malaria control and prevention at least during the elimination phase in an underdeveloped region. Elevation above sea level and precipitation during spring and summer were found to be the other important, respectively, time-invariant and time-dependent variables associated with decreasing and increasing malaria transmission. Indoor residual spraying and the use of insecticide-treated mosquito nets were not found to be effective in decreasing malaria transmission in the elimination phase.

**Conclusions:**

The introduction of electricity to a rural community does not guarantee an absolutely good effect on the reduction of malaria transmission.

## Background

Malaria is one of the most important challenges to global public health. On 2015, the annual number of deaths caused by malaria is estimated to be about 438,000 (range 236,000–635,000), with 306,000 (range 219,000–421,000) of deaths occurring in children under 5 years [1]. In Iran, based on the latest estimations, about 15 % of the population is at risk of malaria transmission, with 5 % of the population (about 3.8 million people) considered to be at high risk, and up to 90 % of malaria cases are reported from only three provinces in the southeast of Iran, namely Hormozgan, Sistan-va-Baluchestan and Kerman [2]. Based on the 2009 World Health Organization’s malaria report, the elimination phase of Iran’s malaria control programme was started in 2004. Since then, in a decreasing trend, the number of indigenous malaria cases has reduced from 1847 in 2010 to 479 in 2013. Generally, more than 90 % of the cases have been *Plasmodium vivax* and the rest *Plasmodium falciparum*. In WHO reports, Iran has usually been categorized among the countries with low, geographically-limited malaria transmission and effective malaria programmes [2–7]. In Iran, malaria foci are almost exclusively restricted to rural areas.

The main malaria control measures used in Iran, in the elimination phase, might be outlined as: (i) Active and passive case finding to reduce the time interval between appearance of the signs and symptoms of malaria and the beginning of treatment to 24 h; the annual blood examination rate in Iran is usually less than 5 % [2]. (ii) Extending the coverage of malaria control measures to over 90 % of the high-risk population. (iii) Vector control interventions.

In Iran, vector control interventions are applied in targeted foci [3] and, where applicable, include (i) Indoor residual spraying (IRS); (ii) provision of insecticide-treated mosquito nets (ITN), free of charge for high-risk population; and (iii) larviciding. During the years 2011–2013, the coverage of IRS for the population living in the high-risk areas was increased from 11 to 26 % and then to 36 % [3], and the Ministry of Health distributed sufficient ITNs to protect over 60 % of the population at high risk [3]. However, before 2011, the use of ITN had a coverage of at most 5 % in the high-risk population [2]. Larviciding via the distribution of *Bacillus thuringiensis* in all swamps and domestic fresh water reservoirs, including household water tanks, and via the distribution of larvivorous fishes (especially Gambusia fish) in swamps in the high-risk regions [8].

During the years 2011–2013, the annual budget allocated for malaria control exceeded US$ 4 per capita. Domestic financing for malaria control in 2013 accounted for 59 % of this budget, and the rest was financed by the Global Fund to Fight AIDS, Tuberculosis and Malaria [3]. All malaria diagnosis and treatment services are free of charge in the public sector, and all suspected cases undergo scrutinized laboratory tests including either microscopic examination of peripheral blood smear or rapid diagnostic tests (RDT) and only confirmed cases receive anti-malarials. Uncomplicated confirmed falciparum malaria cases receive artesunate + sulfadoxine-pyrimethamine + primaquine; and severe falciparum cases receive artesunate or quinine + doxycycline. For confirmed vivax malaria cases, chloroquine + primaquine are prescribed [3].

When studying the environmental factors other than the meteorological ones (such as the precipitation, humidity, and temperature), most researchers usually focus on the socioeconomic factors and not on the developmental ones, even though disentangling these two from each other is sometimes not so easy. In the studies which focus on the socioeconomic factors, the sampling units are usually households or individuals, while for studying the effects of different aspects of development, such as the development of electricity networks or networks of roads, on malaria control, the appropriate sampling units are usually communities, whether small or large [9–13].

The number of studies that have addressed the role of development on malaria control is small. Today, the common notion with regard to the effect of development on malaria is that investment in approaches addressing economic development and improvements in socio-economic status significantly reduce the malaria burden in deprived underdeveloped communities, not only in the long-term but also, in many situations, in short-term [9, 14–19]. There is a common saying among malaria experts in Iran that “when electricity comes, malaria goes”. The main objective of this study was to measure the effects of electricity network development on malaria transmission in malaria foci of the rural regions in southeastern Iran, attempting to disentangle the effects of this development from other routine control measures, such as IRS, and ITNs.

## Methods

The sampling units in this study were rural communities (not individuals), and the words ‘village’ and ‘rural community’ have been used interchangeably. In fact, by using the word ‘village’, the main purpose is to speak about the rural population, i.e., the people living in a well-defined geographic location close to each other and together composing the population of a village.

The villages selected for this study were the ones located in Sistan-va-Baluchestan province in southeastern Iran. The study was implemented during 2010 and the data were collected retrospectively using data banks of the health sector, the offices of the local electricity network and Islamic Republic of Iran’s Meteorological Organization.

Three districts which took part in this study included Chabahar, Nikshahr and Saravan. In each district at least 40 villages were selected, 20 with malaria transmission and the 20 without malaria transmission during 2009. Though equality of the number of villages with regard to malaria transmission was correct for the year 2009, this was not correct for the first 4 years of the study (i.e., 2005–2008), even though their numbers in the two categories (i.e., with and without malaria transmission) remained comparable. There were no other selection criteria and the villages were not matched for any of their characteristics.

The time span of the study was the years 2005–2009. The information for each village was gathered retrospectively using a three-page questionnaire, especially designed for the study. In each of the three districts involved in the study, a health officer, adept in the local language and familiar with the geography of the area, gathered the information about the villages.

The information for the following variables was gathered on an annual basis, i.e., each year separately (for 5 years):

- Monthly precipitation (usually rainfall) from meteorological stations of districts. In the analysis the data were briefed to rainfall in spring and summer and rainfall in autumn and winter).
- Annual number of malaria cases,
- The year of introducing electric power to each village during the study period, and
- The years when IRS had been implemented in the village.

For the above variables, each village was given a code of 1 for having the facility (electricity, IRS, etc.) and nil (0) for otherwise. Therefore, if electricity had reached a village in 2007, the code of that village for electricity were recorded as nil (0) for the years 2005 and 2006 and as (1) for the next 3 years of the study (years 2007–2009).

It might be worth mentioning that IRS as a ‘control measure of malaria transmission’ is usually implemented only on the condition that at least 85 % of the village population cooperates with the spraying teams. These activities are totally free of charge and the insecticides used have residual effect for at least 3 months. If implemented, these sprayings are biannual activities performed once before the beginning of the malaria transmission period, i.e., late February (or early March) and once more in the midst of the transmission period, usually before the end of summer’s heat climax in august.

The following information about each village was gathered only for the year 2009 and was generalized to all the previous years, i.e., it has been presumed that there had not been so much change in the rural community and their living conditions with regard to these variables during the study period.

1. With respect to the availability of health services, each village might be categorized in one of the following groups: main, satellite, or mobile teams. ‘main village’ refers to a village with ‘health house’ facilities, and at least one (and usually two) educated health worker(s). These health workers provide health services not only to the population of the main village but also to the residents of three to seven other villages, known as the ‘satellite villages’ located within a range of about 5 km away from the main village. In a stepwise manner, every three to five health houses are under the supervision of a ‘Rural Health Centre’.
  The term ‘Mobile teams’ refers to health teams in charge of providing ‘Primary Health Care’ (PHC) services to the residents of hard-to-reach small villages and nomads. These teams continuously and regularly travel in the fields and visit those in need of their services, such as children and pregnant women.
2. Larviciding activities: these activities are usually in the form of distributing *B. thuringiensis* and larvivorous fishes and educating people as to the management of potential larvae breeding sites within and around their living places [8, 20].
3. The village population (according to the latest annual local census in each village).
4. The number of households in each village.
5. The distance of the village from the nearest malaria diagnosis and treatment post: these posts (also known in the region as ‘Passive Posts’) are health facilities especially dedicated to the diagnosis and treatment of malaria. These posts are composed of a microscopy laboratory and a technician and one or two health workers whose main job is to follow up and care for malaria cases in the region. In areas that the PHC network has not been developed well, these posts usually are present and manage malaria in the region in the villages located within a range of at least 5 km. Where the PHC coverage has been developed and Rural Health Centres and health houses are well-established, these ‘passive posts’ usually work as one of the units of the Rural Health Centre.
6. The elevation of the villages above sea level (using the Global Positioning System sets).
7. The proportion of houses with appropriate net window.
8. The proportion of households that use ITNs appropriately.

### Analysis

The data were analysed using Stata version 11 (Stata Corp LP, College Station, TX). In the analysis of the data, descriptive tables and statistical tests were used. Independent sample t-test was used to compare means and proportions between the villages with and those without malaria transmission, and Pearson’s correlation coefficient was used for studying the relationship between ‘elevation above sea level’ and ‘proportion of houses with appropriate net window’. Fisher’s exact test (two-sided) was used to study associations in contingency tables with scarce data.

For multivariate analysis, panel data regression was used. This type of data analysis was used because the dataset, which was a combination of time series and cross sections, had the properties of panel data, i.e., was composed of two or more observations on many units [21]. Panel data is a dataset in which the behaviour of entities is observed across time. Panel data allows for controlling of variables that change over time, i.e., it accounts for individual heterogeneity [21–23]. In the present study, the main dependent variable was mean annual number of malaria cases during the 5 years of the study period and the main independent variable was presence or absence of electricity supply to villages. The other variables were measured mostly for the sake of controlling their effects as confounders. To decide between fixed or random effect models, Hausman test was used. It basically tests whether the unique errors are correlated with the regressors, the null hypothesis is that they are not [21, 24]. Fixed-effect models are designed to study the causes of changes within an entity (in this study, a village). A time-invariant attribute cannot cause such a change, because it is constant for each person. If the researcher has some reason to believe that differences across entities have some influence on their dependent variable, then they should use random effect [21, 24].

The results of both random effects and fixed effects models accompanied by the results of Hausman tests have been reported; however, in the absence of evidence for significant bias, the random effects model was preferred, because it explicitly included the time invariant variables, i.e., the ‘elevation above sea level’, the population size, and the ‘proportion of houses with appropriate net window’.

For two of the time invariant variables, i.e., the ‘number of households in a village’ and ‘elevation above sea levels’, the squares of the one hundredths of the original values were used in regression analysis. The original values were divided by 100 to avoid very small coefficients in the model and the results were squared because it fitted a much better model.

## Results

In total, 122 villages (rural communities) were included in the study. The most populated village, with 3012 people, was composed of 621 households and the least populated one, with 38 people, had 11 households. Out of the 122 villages, 42 villages were located in the territory of Chabahar district, 40 in the territory of Saravan, and the rest in the territory of Nikshahr. Because the regions under study were in the pre-elimination phase of malaria control, none of the annual number of cases in any of the villages under study exceeded five (Table 1).

**Table 1:** Average annual number of malaria cases and number of households by transmission situation and year of observation (total number of villages 122)

Year	Malaria transmission	Number of villages	Household numbers				Annual malaria cases			
			Mean (standard deviation)	10th percentile	Median	90th percentile	Mean (standard deviation)	10th percentile	Median	90th percentile
2005	No	46	49.6 (31.2)	13	46	98				
	Yes	76	89.4 (118.6)	18	57.5	151	1.6 (0.8)	1	1	3
2006	No	65	49.2 (30.6)	15	45	92				
	Yes	57	103.1 (133.4)	21	64	256	1.7 (0.8)	1	1	3
2007	No	43	43.2 (30.8)	13	37	81				
	Yes	79	91.3 (115.5)	20	58	149	1.7 (0.8)	1	2	3
2008	No	59	60.8 (80.3)	15	49	81				
	Yes	63	87.1 (110.1)	16	58	149	1.8 (0.8)	1	2	3
2009	No	63	68.9 (82.7)	16	50	109				
	Yes	59	80.5 (111.2)	15	56	123	1.2 (0.4)	1	1	2

In Tables 2 and 3, on an annual basis, the descriptive features of the villages with malaria transmission have been compared with those without malaria transmission. In Table 2, even though all the years showed a statistically-significant difference between malarious and non-malarious villages with regard to IRS, there was no significant effect for this intervention in multivariate analysis (Model No. 4, Table 4). Table 2 shows other, less consistently statistically-significant, findings (such as the difference between malarious and non-malarious villages with regard to ‘availability of health services’ in 2005 or ‘availability of electricity’ in 2006), which were not confirmed in multivariate analysis (Table 4).

**Table 2:** Distribution of some of the important variables by years of observation and transmission of malaria in the villages under study, Sistan-va-Baluchestan province, Iran (total number of villages 122)

Year	Malaria transmission	Availability of electricity			Indoor residual spraying			Precipitations during spring and summer			Category of the village with respect to the availability of health services^a^			
		Not available no. (%)	Available no. (%)	P value*	Not sprayed no. (%)	Sprayed no. (%)	P value*	No rain no. (%)	Rain no. (%)	P value*	Main no. (%)	Satellite no. (%)	Mobile team no. (%)	P value*
2005	Not	9 (50.0)	37 (35.6)	0.295	39 (42.9)	7 (22.6)	0.054	18 (31.0)	28 (43.8)	0.191	9 (36.0)	29 (49.2)	8 (21.1)	0.018
	Yes	9 (50.0)	67 (64.4)		52 (57.1)	24 (77.4)		40 (69.0)	36 (56.2)		16 (64.0)	30 (50.8)	30 (78.9)	
2006	Not	13 (86.7)	52 (48.6)	0.006	52 (59.1)	13 (28.2)	0.045	50 (51.0)	15 (62.5)	0.366	9 (36.0)	31 (52.5)	25 (65.8)	0.073
	Yes	2 (13.3)	55 (51.4)		36 (40.9)	21 (61.8)		48 (49.0)	9 (37.5)		16 (64.0)	28 (47.5)	13 (34.2)	
2007	Not	7 (53.8)	36 (33.0)	0.217	38 (44.7)	5 (13.5)	0.001	0 (0)	43 (35.2)	–	5 (20.0)	22 (37.3)	16 (42.1)	0.194
	Yes	6 (46.2)	73 (67.0)		47 (55.3)	32 (86.5)		0 (0)	79 (64.8)		20 (80.0)	37 (62.7)	22 (57.9)	
2008	Not	4 (80.0)	55 (47.0)	0.196	52 (58.4)	7 (21.2)	0.000	59 (48.4)	0 (0)	–	11 (44.0)	26 (44.1)	22 (57.9)	0.360
	Yes	1 (20.0)	62 (53.0)		37 (41.6)	26 (78.8)		63 (51.6)	0 (0)		14 (56.0)	33 (55.9)	16 (42.1)	
2009	Not	1 (33.3)	62 (52.1)	0.610	45 (60.8)	18 (37.5)	0.016	51 (52.0)	12 (50.0)	1.000	14 (56.0)	32 (54.2)	17 (44.7)	0.575
	Yes	2 (66.7)	57 (47.9)		29 (39.2)	30 (62.5)		47 (48.0)	12 (50.0)		11 (44.0)	27 (45.8)	21 (55.3)	

* Two-sided Fisher exact test

^a^For a description about the categories (main village; satellite village and mobile team villages) please refer to “Methods” section

**Table 3:** Comparison of the mean values of some of the variables between villages with and those without malaria transmission on a yearly basis, using independent sample t-test ; Sistan-va-Baluchestan province, Iran, (total number of villages 122)

Year	Elevation above sea level (meter)			Percent of houses with nets on windows			Number of households			Proportion of households using ITN			Distance from the nearest malaria diagnosis and treatment post (Kilometer)		
	Villages without transmission	Villages with transmission	P value (*t*-test)	Villages without transmission	Villages with transmission	P value (t-test)	Villages without transmission	Villages with transmission	P value* (t-test)	Villages without transmission	Villages with transmission	P value (t-test)	Villages without transmission	Villages with transmission	P value* (t-test)
	Number mean (SD)	Number mean (SD)		Number mean (SD)	Number mean (SD)		Number mean (SD)	Number mean (SD)		Number mean (SD)	Number mean (SD)		Number mean (SD)	Number mean (SD)	
2005	46 728.4 (577.7)	76 567.9 (564.3)	0.133	46 35.1 (36.1)	76 28.9 (33.3)	0.337	46 49.6 (31.2)	76 89.4 (118.6)	0.006	46 42.7 (37.1)	76 32.2 (34.3)	0.115	46 13.4 (12.8)	76 12.6 (13.3)	0.732
2006	65 732.4 (564.1)	57 509.9 (563.3)	0.031	65 33.8 (34.1)	57 28.3 (34.7)	0.385	65 49.2 (30.6)	57 103.1 (133.4)	0.004	65 42.4 (35.8)	57 29.1 (34.3)	0.038	65 14.5 (14.9)	57 11.1 (10.5)	0.145
2007	43 812.0 (581.4)	79 528.5 (545.4)	0.008	43 41.2 (34.9)	79 25.8 (33.0)	0.016	43 43.2 (30.8)	79 91.3 (115.5)	0.000	43 47.2 (37.1)	79 30.2 (33.6)	0.011	43 14.1 (12.3)	79 12.2 (13.5)	0.427
2008	59 871.6 (536.4)	63 400.7 (511.1)	0.000	59 45.3 (34.8)	63 18.0 (34.4)	0.000	59 60.8 (80.3)	63 87.1 (110.0)	0.132	59 44.7 (38.1)	63 28.2 (31.4)	0.009	59 15.2 (15.3)	63 10.8 (10.2)	0.068
2009	63 629.0 (576.1)	59 627.8 (573.3)	0.990	63 30.6 (33.4)	59 31.9 (35.7)	0.836	63 68.7 (82.7)	59 80.5 (111.2)	0.509	63 36.7 (36.4)	59 35.7 (35.1)	0.875	63 14.5 (15.2)	59 11.2 (10.2)	0.155

* Independent sample t-test was done with unequal variances assumption

**Table 4:** Multivariate panel data regression analysis of the relationship between the annual number of malaria cases and villages characteristics, Sistan-va-Baluchestan province; Iran, (total number of villages 122)

Models^a^	Variables under study	Random effect model	P value	Fixed effect model	P value	Hausman test
		Coefficient (95 % CI)		Coefficient (95 % CI)		P value
Model no. 1	Electrification	0.31 (0.01 to 0.61)	0.040	0.10 (−0.31 to 0.50)	0.643	0.0937
	Square of the one hundredth of the number of the households in a village	0.05 (0.03 to 0.07)	0.000	Omitted	–	
	Precipitations during spring and fall (1 = yes; 0 = no)	0.24 (0.10 to 0.39)	0.001	0.26 (0.11 to 0.42)	0.001	
	Square of proportion of houses with appropriate net window	−0.80 (−1.16 to −0.44)	0.000	Omitted	–	
Model no. 2	Electrification	0.22 (−0.07 to 0.52)	0.139	0.10 (−0.31 to 0.50)	0.643	0.4143
	Square of the one hundredth of the number of the households in a village	0.05 (0.03 to 0.07)	0.000	Omitted	–	
	Precipitations during spring and fall (1 = yes; 0 = no)	0.25 (0.10 to 0.40)	0.001	0.26 (0.11 to 0.42)	0.001	
	Square of every hundred meter of elevation above sea level	−0.05 (−0.07 to −0.03)	0.000	Omitted	–	
Model no. 3	Indoor residual spraying	0.31 (0.13 to 0.49)	0.001	0.09 (−0.14 to 0.31)	0.457	0.0045
	Electrification	0.30 (0.01 to 0.58)	0.044	0.09 (−0.31 to 0.50)	0.656	
	Square of the one hundredth of the number of the households in a village	0.05 (0.03 to 0.07)	0.000	Omitted	–	
	Precipitations during spring and fall (1 = yes; 0 = no)	0.24 (0.09 to 0.39)	0.001	0.26 (0.11 to 0.41)	0.001	
	Square of proportion of houses with appropriate net window	−0.62 (−0.97 to −0.27)	0.001	Omitted	–	
Model no. 4	Percent of households that use ITN	−0.003 (−0.006 to 0.000)	0.032	Omitted	–	0.0294
	Square of the one hundredth of the number of the households in a village	0.05 (0.03 to 0.06)	0.000	Omitted	–	
	Precipitations during spring and fall (1 = yes; 0 = no)	0.22 (0.07 to 0.36)	0.003	0.26 (0.11 to 0.41)	0.001	
	Square of proportion of houses with appropriate net window	−0.70 (−1.10 to −0.32)	0.000	Omitted	–	

^a^The dependent variable in all instances has been the number of malaria cases during each year

In Table 3, in years 2006–2008, there were statistically significant differences between malarious and non-malarious villages with regard to the ‘proportion of the households using ITN’; however, these were not confirmed in the more comprehensive regression analysis (Table 4). The distance of the villages from the nearest malaria diagnosis and treatment post showed no statistically significant association with malaria transmission, neither in univariate nor in multivariate analysis. The other three variables in Table 3—i.e., elevation above sea level, proportion of houses with appropriate net window and number of households in each village—showed statistically significant association with malaria transmission (each for 2 or 3 years of the study), and their role in malaria transmission were also assessed as significant in the more comprehensive regression analysis (Table 4, Model No. 1 and Model No. 2).

Table 4 reports the results of the panel data regression analysis for fixed effect and random effect models and the results of Hausman test. To avoid the effect of collinearity, between the ‘elevation above sea level’ and ‘the proportion of houses with appropriate net window’, a separate model was fitted for elevation above sea level (Table 4, Model No. 2). The Pearson’s correlation coefficient between the ‘elevation above sea level’ and the ‘proportion of houses with appropriate net window’ was 0.8524 (P value = 0.0000).

Based on the results of Hausman test, models containing either IRS or the proportion of households that use ITNs, were not suitable to be fitted in a random effect model; and in the fixed effect model, the effect of the first one was not significant statistically, and the second one was a time-invariant variable (Table 4, Models No. 3 and 4). There was no association between the mean number of annual malaria cases and the availability of health services in none of the regression models.

The only variable that its effect was significant in all regression models was the binomial variable ‘precipitation during spring and fall’ (‘1’ for occurrence of precipitation and ‘0’ for no precipitation).

## Discussion

In the interpretation of the results of analysis, especially regression analysis (Table 4), it should be kept in mind that the study area is in the malaria elimination phase and the mean annual number of malaria cases during the study period barely comes to 1.0 (Table 1). Second, it is worth notifying that due to design of the study, which has repeated measurements on the same sampling units during 5 consecutive years, the results of analysis for each year (and the reported P values), presented in Tables 2 and 3, might not be discretely interpretable on an annual basis. These data have been presented mostly for describing the distribution of attributes, exposures and traits in the sampling units (villages) in the course of time.

The data collection method of some of time-invariant variables such as ‘The proportion of houses with appropriate net window’, ‘the proportion of household using bed nets’, and ‘larviciding activities’ have to be referred to as one of the most important limitations of the present study. The main reason these data were not gathered on an annual basis was that there was no log book or registry for producing reliable data about them. Hence, either their effects must be ignored in the study or they have to be entered as time-invariant variables. Since these variables are very important covariates in every study on vector-borne diseases such as malaria, the second option was chosen, i.e., they were entered as time-invariant variables and presumed that their size has been relatively constant during the 5 years in the time span of the study.

One of the main purposes of implementation of this study was studying the effect of one of the most important features of development in the rural regions, i.e., the electricity. A good (reducing) effect of electricity on malaria transmission was expected to be seen, while contrary to this expectation, it seems that the introduction of electricity led to an increase in the occurrence of malaria, i.e., seemingly the presence of electricity increases the average number of annual malaria cases about 0.31 (95 % CI 0.010–0.61) (Model No. 1, Table 4). These finding, however, are not so robust statistically, e.g., in Model No. 2, electrification has an insignificant coefficient in both the random effect model and the fixed effect model; and in Model No. 3, electrification has an insignificant coefficient in the fixed effects model, and the Hausman test rejects the random effect model. One might suggest that the overall finding should be that after controlling for other variables, changes in electrification are not associated with changes in malaria outcomes over time. In a cohort study of various anti-mosquito interventions in Pakistan, it was found that an electric ceiling fan running at high speed significantly reduced the total catches of blood-fed Culicine mosquitoes [25, 26]. However, it did not significantly reduce the total catches of blood-fed Anopheline mosquitoes. These studies suggest that Anopheline mosquitoes may be more tolerant than Culicine mosquitoes of air turbulence [26]. In another case control study in Peru, electricity did not have any role as a risk factor either in univariate or in multivariate analysis [27].

‘Number of households in a village’, almost like elevation above the sea level, is not amenable to sudden annual changes; and in a stable social situation, it acts most likely as a time-constant variable. In univariate analysis and on an annual basis, the mean number of households in the villages with malaria transmission was higher in the first 4 years of the study (Table 3); and in panel data regression analysis combining all 5 years of the study together, the coefficient for this variable was 0.05 (95 % CI 0.03–0.07) (Table 4, Model No. 1). As it was mentioned in the “Methods” section, the numbers of households were divided by 100 to avoid very small coefficients in the model and the result was squared because it fitted a much better model. The relationship between human population structure and the distribution of malaria has not been considered as a reportable variable in most epidemiologic studies of malaria; however, this finding has not been unique to our study; and in some other studies, similar situations have been reported. For instance, in a study conducted in southeast Nigeria, the risk of malaria occurrence was higher in the urban areas than in the rural ones [10]. In a study in Cameroon, the spatial pattern of the urban malaria occurrences displayed a complex combination of population density gradients and socio-environmental factors, i.e., those features differentiating rural areas and urban ones combined with population density were determining the effectiveness of malaria control measures [28].

The elevation above sea level as a time-invariant variable also revealed interesting relationships (Table 3). Each year (except 2009), the mean elevation of villages with active transmission of malaria was lower than that of villages without transmission. In addition, in panel data regression analysis using random effect model, for the square of every 100 m elevation above sea level, the average number of annual malaria cases decreased about 0.05 (Table 4). The effect of weather and elevation above sea level on the occurrence of most vector-borne diseases is a well-known fact and has been mentioned in many other studies [29–32].

The information about monthly precipitation was gathered from the nearest meteorological station. This variable was the only one that showed statistically significant relationship both in fixed effect and in random effect models in panel data regression analysis (Table 4). In all four models, there was a strong positive relationship between precipitation during spring and summer and the average annual number of malaria cases, i.e., precipitation during spring and summer increased the average annual number of malaria cases about 0.24–0.26 (Table 4). The mechanism of action of seasonal precipitation and humidity on vector density and malaria transmission is a well-known fact of the type of common knowledge [33–38].

One of the most important variables in this study was indoor residual spraying (IRS). Details about the implementation of this biannual activity were described in the “Methods” section. Based on statistical findings in this study, IRS, had no significant effect in the fixed effect model (P value = 0.457), while in the random effect model, it showed a relatively strong effect (P value = 0.001) (Table 4, Model No. 3); however, since based on the result of Hausman test (P value = 0.0181), the fixed effect model was a better suit for it, the common speculation is that, from the statistical point of view, IRS might not be considered as an effective malaria control measures, at least in the elimination phase and in this study setting. However, based on studies in other situations, the role of IRS in controlling and preventing malaria is a well-established fact almost at the level of common knowledge [13, 39, 40].

Except for 2009, during each year of the study, the mean proportion of houses with appropriate net window was lower in villages with transmission than in villages without transmission (Table 3). Also, in panel data regression analysis, a relatively large coefficient for the square of the proportion of houses with appropriate net window shows the same direction and was confirming the results of mono-variant analysis (Table 4). The mechanism of action of mounting of nets on windows is thoroughly known and might be considered of the type of common knowledge [33].

With a look at Table 3, it might be found easily that on an annual basis, the average of the ‘proportion of households using ITN’ is lower in villages with malaria transmission than those without malaria transmission. This has been correct for all the years of the study perhaps except 2009, which the two proportions are almost equal. However, in multivariate analysis, since based on the results of the Hausman test, the random effect models containing this variable were not appropriate for studying the effects of the variables; and since in the fixed effect model, no time-invariant variable might be included and studied, we should conclude that from the statistical point of view, the effect of this intervention in the study population might not be considered significant (Table 4, Model No. 4). The importance of availability of ITN and the quality of its use have been reminded in many other studies but usually at an individual level and not in a population study [9, 12, 40–42].

Among the time-invariant variables, the village distance from the nearest malaria diagnostic post as a time-invariant variable was not associated with malaria transmission, neither in univariate nor in regression analysis. However, in most other studies, the usual effect of ease of access to health facilities has been a decrease in the number of cases [43].

In this study, it is tried to clarify the effect of electricity network development as one of the most important interventions in remote underdeveloped areas in preventing and controlling malaria in a region that was in the primary stages of malaria elimination. In addition to this and in order to control other cofactors, we also studied the effect of other routine interventions such as indoor house sprayings, use of ITNs, and the effect of coverage of health services. What was found was far from expectation, i.e., the consequences of the introduction of electricity to a rural community, if not harmful, without any doubt might not be assessed as helpful at least within the framework of the present study setting. This finding, albeit contrary to earlier predictions, increases the value of the present study, because it implies that perhaps reliance on some features of development is more than that they deserve and because perhaps both the people and the health sector have to learn how to use the new features of development.

## Conclusions

Based on the findings in this study, regardless of some exceptions (the use of ‘window nets’), the factors that showed the most constant and reliable effects on malaria control were those that are out of health sector control, i.e., annual precipitation, elevation above sea level, and the population of villages, and those factors that health sector usually relies on as the most effective tools in the control and prevention of malaria were either of no obvious effect or even harmful; factors such as regular ‘indoor residual spraying’, distribution of ITN (even free of charge) and electrification of villages. These findings teach both the health sector and people fighting malaria to prohibit overestimation of the effect of development (in any aspect) on malaria control. Malaria control, first and foremost, depends on the beliefs, habits, health knowledge, health behaviour, and capabilities of the people who are living with malaria and then, in the next stages, on the facilities.

## Abbreviations

ITN: insecticide-treated mosquito net
IRS: indoor residual spraying
RDT: rapid diagnostic tests

## References

[1] WHO. World Malaria Report 2015. Geneva: World Health Organization; 2015.

[2] WHO. World Malaria Report 2009. Geneva: World Health Organization; 2009.

[3] WHO. World Malaria Report 2014. Geneva: World Health Organization; 2014.

[4] WHO. World Malaria Report 2013. Geneva: World Health Organization; 2013.

[5] Ostovar A, Raeisi A, Haghdoost AA, Ranjbar M, Rahimi A, Sheikhzadeh K (2012). Lessons learnt from malaria epidemics in the Islamic Republic of Iran. East Mediterr Health J.

[6] Kummer T. Together for health: the Islamic Republic of Iran working with the Global Fund and UNDP against HIV/AIDS, tuberculosis and malaria. UN Development Assistance Framework (UNDAF); 2012.

[7] WHO. World Malaria Report 2010. Geneva: World Health Organization; 2010.

[8] Vatandoost H, Mashayekhi M, Abaie MR, Aflatoonian MR, Hanafi-Bojd AA, Sharifi I (2005). Monitoring of insecticides resistance in main malaria vectors in a malarious area of Kahnooj district, Kerman province, southeastern Iran. J Vector Borne Dis.

[9] Yadav K, Dhiman S, Rabha B, Saikia P, Veer V (2014). Socio-economic determinants for malaria transmission risk in an endemic primary health centre in Assam, India. Infect Dis Poverty.

[10] Onwujekwe O, Uzochukwu B, Dike N, Okoli C, Eze S, Chukwuogo O (2009). Are there geographic and socio-economic differences in incidence, burden and prevention of malaria? A study in southeast Nigeria. Int J Equity Health.

[11] Onwujekwe O, el Malik F, Mustafa SH, Mnzavaa A (2006). Do malaria preventive interventions reach the poor? Socioeconomic inequities in expenditure on and use of mosquito control tools in Sudan. Health Policy Plan.

[12] Somi MF, Butler JR, Vahid F, Njau J, Kachur SP, Abdulla S (2007). Is there evidence for dual causation between malaria and socioeconomic status? Findings from rural Tanzania. Am J Trop Med Hyg.

[13] Al-Taiar A, Assabri A, Al-Habori M, Azazy A, Algabri A, Alganadi M (2009). Socioeconomic and environmental factors important for acquiring non-severe malaria in children in Yemen: a case-control study. Trans R Soc Trop Med Hyg.

[14] Yang HM (2001). A mathematical model for malaria transmission relating global warming and local socioeconomic conditions. Rev Saude Publica.

[15] Wang SQ, Li YC, Zhang ZM, Wang GZ, Hu XM, Qualls WA (2014). Prevention measures and socio-economic development result in a decrease in malaria in Hainan, China. Malar J.

[16] Sonko ST, Jaiteh M, Jafali J, Jarju LB, D’Alessandro U, Camara A (2014). Does socio-economic status explain the differentials in malaria parasite prevalence? Evidence from the Gambia. Malar J.

[17] Njau JD, Stephenson R, Menon M, Kachur SP, McFarland DA (2013). Exploring the impact of targeted distribution of free bed nets on households bed net ownership, socio-economic disparities and childhood malaria infection rates: analysis of national malaria survey data from three sub-Saharan Africa countries. Malar J.

[18] Lowe R, Chirombo J, Tompkins AM (2013). Relative importance of climatic, geographic and socio-economic determinants of malaria in Malawi. Malar J.

[19] Dickinson KL, Randell HF, Kramer RA, Shayo EH (2012). Socio-economic status and malaria-related outcomes in Mvomero District, Tanzania. Glob Public Health.

[20] Gezelbash Z, Vatandoost H, Abai MR, Raeisi A, Rassi Y, Hanafi-Bojd AA (2014). Laboratory and field evaluation of two formulations of *Bacillus thuringiensis* M-H-14 against mosquito larvae in the Islamic Republic of Iran, 2012. East Mediterr Health J.

[21] McManus PA. Introduction to regression models for panel data analysis, Workshop in methods, USA: Indiana Unviersity. Slides. 2011. http://www.indiana.edu/~wim/docs/10_7_2011_slides.pdf. Accessed 20 July 2015.

[22] Bell A, Jones B (2015). Explaining fixed effects: random effects modeling of time-series cross-sectional and panel data. Polit Sci Res Methods.

[23] Agresti A (2015). Foundations of linear and generalized linear models.

[24] Torres-Reyna O. Panel data analysis fixed and random effects using stata (v. 4.2). Slides. Princeton: Princeton University, 2007. http://www.princeton.edu/~otorres/Panel101.pdf. Accessed 20 July 2015.

[25] Hewitt SE, Farhan M, Urhaman H, Muhammad N, Kamal M, Rowland MW (1996). Self-protection from malaria vectors in Pakistan: an evaluation of popular existing methods and appropriate new techniques in Afghan refugee communities. Ann Trop Med Parasitol.

[26] Croft AM (2010). Malaria: prevention in travellers. BMJ Clin Evid.

[27] Rosas-Aguirre A, Ponce OJ, Carrasco-Escobar G, Speybroeck N, Contreras-Mancilla J, Gamboa D (2015). *Plasmodium vivax* malaria at households: spatial clustering and risk factors in a low endemicity urban area of the northwestern Peruvian coast. Malar J.

[28] Ngom R, Siegmund A (2015). The key role of socio-demographic and socio-environmental factors in urban malaria occurrence and control—An illustration using the city of Yaounde. Soc Sci Med.

[29] el Ngom HM, Faye ND, Talla C, el Ndiaye H, Ndione JA, Faye O (2014). Anopheles arabiensis seasonal densities and infection rates in relation to landscape classes and climatic parameters in a Sahelian area of Senegal. BMC Infect Dis.

[30] Midekisa A, Beyene B, Mihretie A, Bayabil E, Wimberly MC (2015). Seasonal associations of climatic drivers and malaria in the highlands of Ethiopia. Parasit Vectors.

[31] Lardeux F, Aliaga C, Tejerina R, Torrez L (2013). Comparison of transmission parameters between *Anopheles argyritarsis* and *Anopheles pseudopunctipennis* in two ecologically different localities of Bolivia. Malar J.

[32] Dambach P, Machault V, Lacaux JP, Vignolles C, Sié A, Sauerborn R (2012). Utilization of combined remote sensing techniques to detect environmental variables influencing malaria vector densities in rural West Africa. Int J Health Geogr.

[33] Warrell DA, Gilles HM (2002). Essential malariology.

[34] Dantur Juri MJ, Estallo E, Almiron W, Santana M, Sartor P, Lamfri M (2015). Satellite-derived NDVI, LST, and climatic factors driving the distribution and abundance of Anopheles mosquitoes in a former malarious area in northwest Argentina. J Vector Ecol.

[35] Srimath-Tirumula-Peddinti RC, Neelapu NR, Sidagam N (2015). Association of climatic variability, vector population and malarial disease in district of Visakhapatnam, India: a modeling and prediction analysis. PLoS ONE.

[36] Bashar K, Tuno N (2014). Seasonal abundance of Anopheles mosquitoes and their association with meteorological factors and malaria incidence in Bangladesh. Parasit Vectors.

[37] Sena L, Deressa W, Ali A (2015). Correlation of climate variability and malaria: a retrospective comparative study, southwest Ethiopia. Ethiop J Health Sci.

[38] Guo C, Yang L, Ou CQ, Li L, Zhuang Y, Yang J (2015). Malaria incidence from 2005 to 2013 and its associations with meteorological factors in Guangdong, China. Malar J.

[39] WHO. Malaria control and elimination: report of a technical review. Geneva: Switzerland; 2008.

[40] Deressa W, Ali A, Berhane Y (2007). Household and socioeconomic factors associated with childhood febrile illnesses and treatment seeking behaviour in an area of epidemic malaria in rural Ethiopia. Trans R Soc Trop Med Hyg.

[41] Raiesi A, Nikpour F, Ansari-Moghaddam A, Ranjbar M, Rakhshani F, Mohammadi M (2011). Baseline results of the first malaria indicator survey in Iran at the health facility level. Malar J.

[42] Mohammadi M, Ansari-Moghaddam A, Raiesi A, Rakhshani F, Nikpour F, Haghdost A (2011). Baseline results of the first malaria indicator survey in Iran at household level. Malar J.

[43] Schoeps A, Lietz H, Sie A, Savadogo G, De Allegri M, Muller O (2015). Health insurance and child mortality in rural Burkina Faso. Glob Health Action.

